# Temperature Variations around Medication Cassette and Carry Bag in Routine Use of Epoprostenol Administration in Healthy Volunteers

**DOI:** 10.1371/journal.pone.0052216

**Published:** 2012-12-27

**Authors:** Yuichi Tamura, Yasuo Nakajima, Yasushi Ozeki, Tomohiko Ono, Makoto Takei, Tsunehisa Yamamoto, Keiichi Fukuda

**Affiliations:** 1 Department of Cardiology, Keio University School of Medicine, Shinjuku-ku, Tokyo, Japan; 2 Development and Medical Affairs Division, GlaxoSmithKline KK, Shibuya-ku, Tokyo, Japan; Vanderbilt University Medical Center, United States of America

## Abstract

**Background:**

According to several treatment guidelines, epoprostenol is an important treatment option for pulmonary arterial hypertension. However, the pharmacokinetic characteristics and poor stability of epoprostenol at room temperature make its administration challenging. We therefore studied temperature fluctuations between the drug administration cassette and atmosphere to promote the safe use of epoprostenol.

**Methods and Findings:**

Five healthy volunteers carried a portable intravenous infusion pump attached to a medication cassette containing saline in a bag during their ordinary activities over 16 days during which the mean atmospheric temperature was 29.6±1.5°C. The temperature around the medication cassette was not less than 25°C on any occasion, and the mean period over 24 h during which the temperature around the cassette exceeded 35°C and 40°C was 96.9±156.4 min and 24.4±77.3 min, respectively. Significant correlations were observed between the temperatures outside the bag and around the cassette, as well as between temperatures around the cassette and of the saline solution in the cassette (r = 0.9258 and 0.8276, respectively). There were no differences in the temperatures outside the bag or around the cassette with respect to the bag material.

**Conclusions:**

Temperatures around a medication cassette and outside the bag containing the medication increase with sunlight exposure. The temperature around cassettes used for administering epoprostenol must therefore be kept low for as long as possible during hot summer conditions to maintain the drug stability.

## Introduction

Pulmonary arterial hypertension (PAH) is a progressive disease characterized by increased pulmonary arterial pressure and pulmonary vascular resistance, which eventually results in death due to right heart failure. The median life expectancy is longer than 7 years, and the 5-year survival rate of PAH patients is 65% [1]. There are effective treatments that target the pathophysiology of PAH, among which the vasodilator prostanoids are the best established [2], [3]. Several oral therapies targeting other pathophysiological mechanisms including endothelin receptor antagonists and PDE5 inhibitors have become available in recent years; these therapies have significantly improved the management and outcomes of these patients. Despite the availability of several oral therapies, epoprostenol remains an important treatment option for PAH patients; several treatment guidelines worldwide recommend epoprostenol for the treatment of class III and IV (class Ia recommendation) PAH patients [4]. Treatment with higher dosages of epoprostenol improves hemodynamics to a greater extent in PAH treatment [5]. However, its pharmacokinetic characteristics and poor stability at room temperature make its administration challenging. Thus, epoprostenol solution is administered as a continuous intravenous infusion via a central venous catheter; it is necessary to use ice packs to keep the temperature of the epoprostenol cassette below 8°C when the reconstituted solution is used beyond 8 h [6]. This need for icepacks for everyday use can cause considerable inconvenience and discomfort for patients. Therefore, a formulation of epoprostenol with higher temperature stability is highly desirable.

However, the maximum and minimum temperatures of the drug cassette and carry bag during routine use, fluctuations in temperature during a routine 24-h period, and the relationships of these variations with atmospheric temperatures have not been investigated. Such information will be useful to both physicians and patients so that necessary precautions are taken to ensure safe and effective use of epoprostenol, whose stability is affected by higher temperatures.

## Materials and Methods

### Study Design

The investigation was performed in cities located in Saitama, Yamanashi, Gifu, Shiga, and Osaka Prefectures from August 11, 2011, to August 25, 2011. Five healthy volunteers carried the CADD-Legacy Pump (Smiths Medical Inc.) in a bag during their daily living activities. The 100-mL medication cassette was filled with physiological saline instead of the usual epoprostenol solution and attached to the CADD-Legacy Pump. However, no interventions were performed on the volunteers, and no samples were gathered. This study does not fall under the category of a clinical trial according to the ethical guidelines for clinical studies of the Ministry of Health, Labour and Welfare, Japan; therefore, ethical committee approval was not required. Despite this, we obtained written informed consent from all volunteers.

### Temperature and Weather Record Sampling

A temperature sensor with a recording function (Temp Tale4, Nihon Sensitech Corp.) was attached to the side of the cassette, and a second sensor was attached to the outside of the bag. Each temperature sensor recorded the temperature at 10-min intervals for 24 h from the start of the study. Five volunteers participated in the study for 24 h continuously on at least 3 occasions. On each occasion, the volunteer recorded the weather of the current day, type of bag containing the pump, activity undertaken every hour, and location (i.e., indoors or outdoors). As indoor conditions are likely to be controlled by air conditioning, to comprehensively assess temperatures, the subjects were required to spend time or undertake activities outdoors for at least 2 consecutive hours per day.

We also investigated the temperatures of the solution in the medication cassette, and their correlation with temperatures around the medication cassette. Three healthy volunteers carried a medication cassette containing saline and a thermometer to record for 24 h continuously on at least three occasions. Additionally, temperature changes outside the bag, around the cassette, and in the solution were compared between when the bag was left outdoors in the sunlight and in the shade.

The temperature sensors were retrieved after the investigation was completed, and the temperatures recorded therein were tabulated. Data for atmospheric temperature and sunshine duration on the days of the investigation were obtained from appropriate meteorological agencies.

### Statistical Analysis

All statistical analyses were performed with SAS software version 9.1.3 (SAS Institute Japan Ltd., Tokyo, Japan). The correlations of temperatures within groups were analyzed using the correlation (CORR) procedure. Regression curves from plotted graphs were calculated using the general linear model (GLM) procedure.

## Results

### Correlation Among the Temperatures at each Site

**Figure 1:**
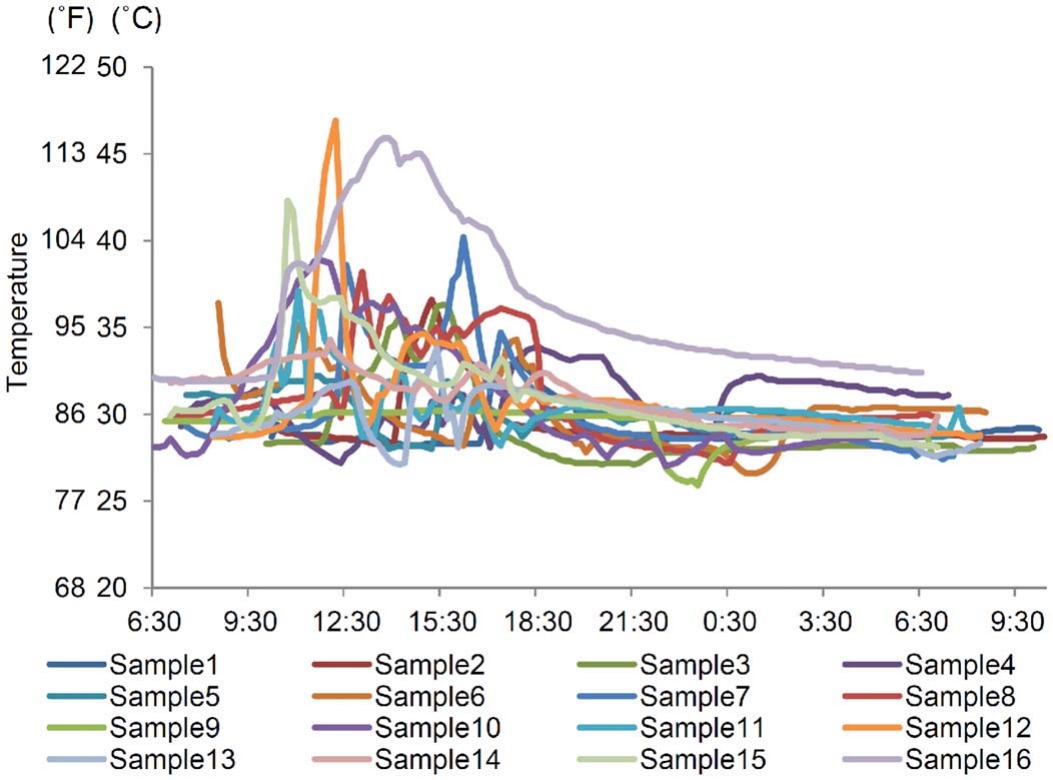
Temperature changes around the medication cassette.

**Figure 2:**
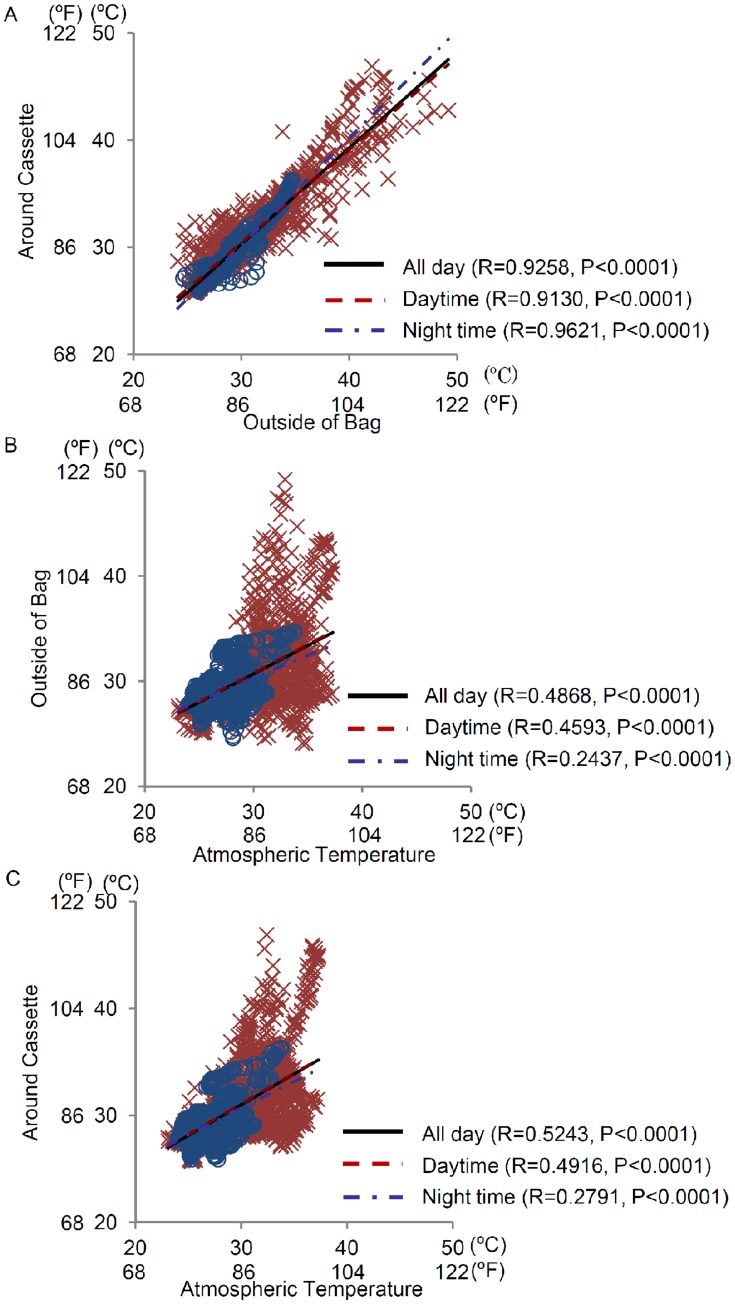
Temperature correlation among outside the bag, around cassette and atmosphere. Correlation between the (A) temperature outside the bag and around the cassette, (B) atmospheric temperature and temperature outside the bag, and (C) atmospheric temperature and temperature around the cassette in daytime (×) and nighttime (○). The relativity of temperatures within groups was analyzed using the CORR procedure. Regression curves from plotted graphs were calculated using the GLM procedure.

**Figure 3:**
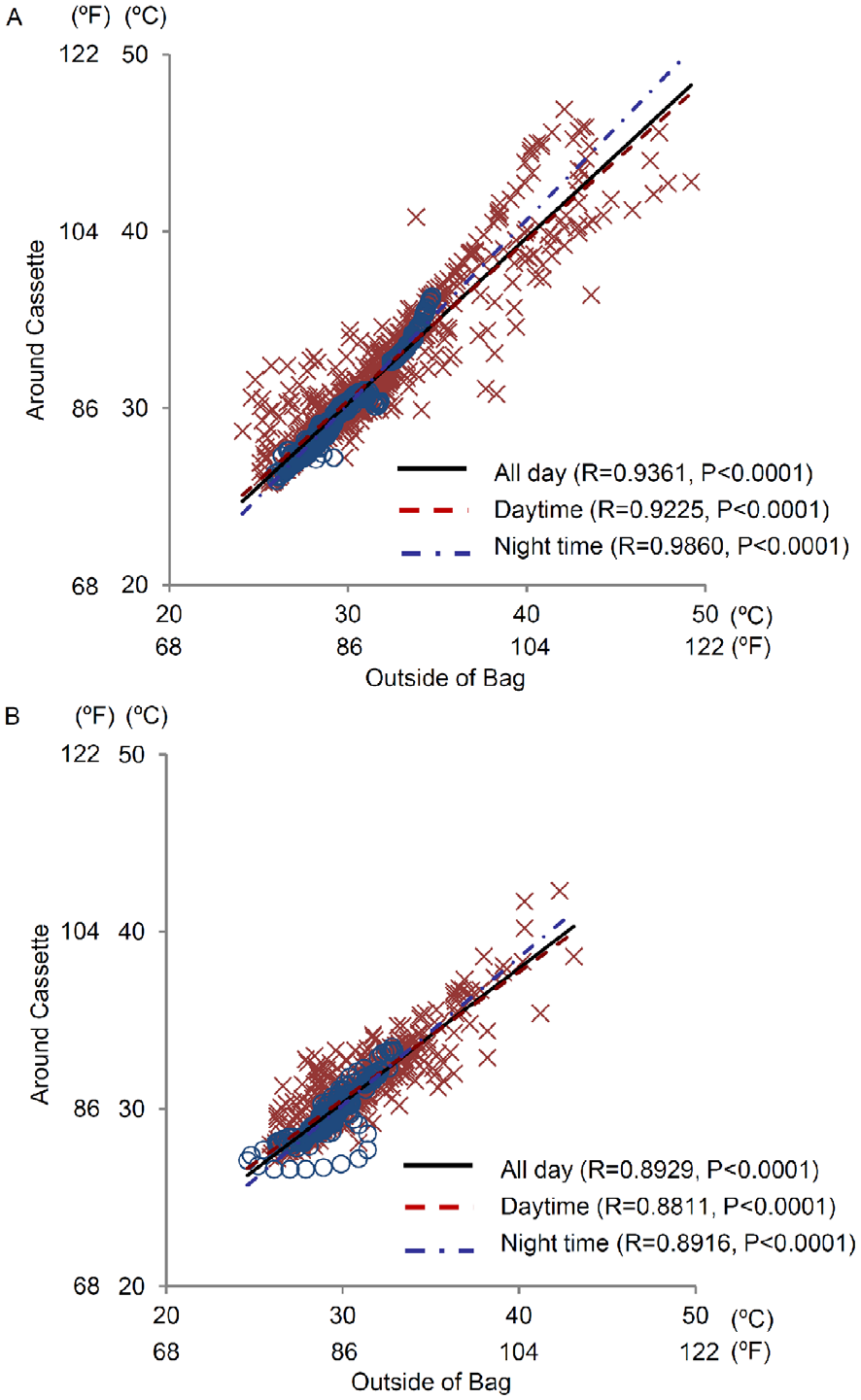
Difference in temperature correlation by material of the bag. Correlations between temperature outside the bag and temperature around the cassette in (A) cloth bags and (B) leather/synthetic leather bags in daytime (×) and nighttime (○). The relativity of temperatures within groups was analyzed using the CORR procedure. Regression curves from plotted graphs were calculated using the GLM procedure.

A total of 16 assessments were performed. The environmental conditions on each occasion of the investigation are shown in Table S1. During the investigation periods, the mean, mean maximum, and mean minimum atmospheric temperatures were 29.6±1.5°C (85.3±2.7°F), 34.6±1.9°C (94.3±3.4°F), and 26.1±1.5°C (79.0±2.7°F), respectively (Table 1). The temperature around the medication cassette did not fall below 25°C (77°F) on any occasion during the investigational period; however, it exceeded 35°C (95°F) on some occasions (Figure 1). During the study period, the mean durations during which the temperature around the cassette exceeded 35°C or 40°C were 96.9±156.4 min and 24.4±77.3 min, respectively. On 4 instances, the temperature around the medication cassette rose rapidly, i.e., the rise in temperature was more than 6°C (11°F) in 20 min. However, on the whole, the temperatures rose slowly, i.e., the temperature increased by 6°C in more than 60 min. Three different temperature correlation analyses were conducted: (1) between the temperatures outside the bag and around the cassette, (2) between the temperature outside the bag and atmospheric temperature, and (3) between the temperature around the cassette and atmospheric temperature. The temperatures outside the bag and around the cassette were positively correlated (r = 0.9258, *P*<0.0001, Figure 2A). Meanwhile, weak correlations were observed between the atmospheric temperature and temperature outside the bag (r = 0.4868 for all days, *P*<0.0001, Figure 2B), and atmospheric temperature and temperature around the cassette (r = 0.5243 for all days, *P*<0.0001, Figure 2C). At one instance in which the temperatures outside the bag and around the cassette increased rapidly, there were no correlations between them, the atmospheric temperature, or the sunshine duration. The changes in the bag and cassette temperatures in shaded areas were similar to those in atmospheric temperature when the bag was left outdoors in the daytime (Figure S1A). When the bags were exposed to sunlight, continuously high temperatures persisted irrespective of the atmospheric temperature (Figure S1B). Additionally, the influence of the material of the bag on the temperature outside the bag and around cassette in daytime was analyzed. However, no differences were observed between synthetic leather, leather, and cloth bags (Figure 3). On one occasion, the temperature around the cassette increased at a greater rate than that outside the bag when left outdoors for more than 2 h. In addition, the temperature around the cassette decreased slower than the temperature outside the bag after returning indoors, although the temperatures around the cassette were very close to or lower than those outside the bag before going outdoors (data not shown).

**Table 1:** Atmospheric temperature and time durations when the temperature around the cassette exceeded 25°C, 35°C, and 40°C.

	Maximum	Minimum	Mean	S.D.
Mean atmospheric temperature (°C/°F)	31.7/89.1	27.0/80.6	29.6/85.3	1.5/2.7
Maximum atmospheric temperature (°C/°F)	37.3/99.1	31.1/88.0	34.6/94.3	1.9/3.4
Minimum atmospheric temperature (°C/°F)	28.7/83.7	23.1/73.6	26.1/79.0	1.5/2.7
Determination time (min)	1450	1450	1450	0
Cumulative time (min) during which the temperature around the cassette exceeded 25°C (77°F)	1450	1450	1450	0
Cumulative time (min) during which the temperature around the cassette exceeded 35°C (95°F)	0	600	96.9	156.4
Cumulative time (min) during which the temperature around the cassette exceeded 40°C (113°F)	0	310	24.4	77.3

### Correlation between Temperatures Around the Cassette and in the Solution

Nine additional assessments were performed to compare temperatures around the cassette and in the solution. During the investigation period, the mean temperatures around the cassette and in the solution were 28.8±1.0°C (83.8±1.8°F) and 29.3±1.0°C (84.7±1.8°F), respectively. The temperature of solution did not fall below 25°C (77°F) on any occasion. Temperature around the cassette therefore correlated positively with those of the solution (r = 0.8276, *P*<0.0001; Figure S2).

The peak temperature of the solution was delayed compared to that outside the bag and around the cassette. Moreover, the temperature decline of the solution was also delayed compared to decreases in temperature around the cassette (Figure S1 A and B). To evaluate how sensitive the temperature was to change at each site, we compared temperature changes every 10 minutes in each segment within the nine additional assessments. The mean change in solution temperature (0.15±0.19°C/0.27±0.34°F) tended to be less than that outside the bag (0.77±1.25°C/1.35±2.25°F, Figure S3).

## Discussion

In this study, the changes in temperature around the cassette and carrying bag during routine daily activities in the hottest period of summer were investigated. Summers in Japan are hot and humid, and the atmospheric temperature exceeds 30°C regularly. In this study, the change in temperature around the cassette was strongly correlated with the temperature outside the bag. There was a high correlation between the temperatures of the solution and around the cassette (r = 0.8276: Figure S2). These results suggested that measurement of temperature around the cassette provided a good representation of the solution temperature inside the cassette bag. In contrast, the cassette temperature and temperature outside the bag were weakly correlated with the atmospheric temperature. Furthermore, when the bag was left outdoors in shaded areas all day long, the temperatures outside the bag and around the cassette tended to follow changes in the atmospheric temperature, although the baseline temperatures were different. However, when the bag was exposed to direct sunlight, temperature of the cassette and temperature outside the bag were considerably higher than the atmospheric temperature; the atmospheric temperature was weakly correlated with the temperatures outside the bag and around the cassette. Moreover, the temperature inside cars can increase significantly in sunlight even in winter [7]. Although, it is possible that patients with PAH with significant functional impairment are likely to spend most of their time indoors where temperatures are controlled by air conditioning, the present findings demonstrate that exposure to sunlight can significantly increase the temperatures of the cassette very quickly. Therefore, additional care should be taken to protect the bag and cassette/pump system from sunlight exposure. Other studies report that the thickness of the cloth of a bag may influence the temperature under radiant light [8]. However, the present results indicate that the bag material does not affect changes in cassette temperature; in this study, there were no significant differences with respect to temperature changes between bags made of cloth, leather, or synthetic leather.

Moreover, mean temperature changes every 10 minutes were smaller for the solution compared to the other three measurement sites. These results suggested that temperature of the medication solution is less influenced by rapidly rising temperatures around the cassette and outside the bag, but also that once the temperature of solution rises with long-term exposure to a high temperature such as that induced by sunlight exposure, the increased temperature is maintained for a prolonged time. This highlights the need to avoid exposure to direct sunlight or minimize any unavoidable exposure to raised atmospheric temperature.

This study was performed in the summer to evaluate the temperatures during the hottest time of the year. However, a similar study in the winter may also be useful since patients may spend most of their time in warm environments and inadvertently come into close contact with various heating sources–the impacts of which were not assessed in the present study.

### Conclusion

The results of this study demonstrate that the cassette temperature and temperature outside the bag are closely correlated and are likely to be higher than the atmospheric temperature in conditions of both shade and direct sunlight. In addition, in Japan during summer, the temperatures of the cassette and carrying bag on the study days remained consistently above 25°C (77°F) and at times, up to 35°C (95°F) or higher. These results suggest that exposure to direct sunlight should be avoided to minimize the risk of medication cassette temperatures exceeding the temperature specifications for intravenous medications that are used for continuous infusion.

## Supporting Information

Figure S1 Temperature changes in (A) shaded and (B) sunny regions. Temperature changes outside the bag, around the cassette and in the solution. They were recorded when the bag was left outdoors in the shade (A) and in the sunny (B) areas.
(TIF)

Figure S2 **Temperature correlation between the solution and around the cassette.** Correlations between daytime (×) and night (○) temperatures around the cassette and in the solution. The relativity of temperatures within groups was analyzed using the CORR procedure. Regression curves from plotted graphs were calculated using the GLM procedure.
(TIF)

Figure S3 **Mean temperature changes over time.** Temperature changes every 10 minutes were analyzed in the solution, around the cassette, outside the bag, and in the atmosphere (0.15±0.19(SD)°C/0.27±0.34°F, 0.37±0.66°C/0.67±1.2°F, 0.77±1.25°C/1.38±2.26°F and 0.32±0.30°C/0.58±0.54°F, respectively).
(TIF)

Table S1 Atmospheric temperature and duration of time in which the temperature was higher than 25, 35 or 40°C around cassette on determination of each sample.
(DOC)
