# Supplementary figures and images for: Temperature Variations around Medication Cassette and Carry Bag in Routine Use of Epoprostenol Administration in Healthy Volunteers
Source: PLoS One. 2012 Dec 27;7(12):e52216. doi: 10.1371/journal.pone.0052216 (PMC3531421; doi:10.1371/journal.pone.0052216)

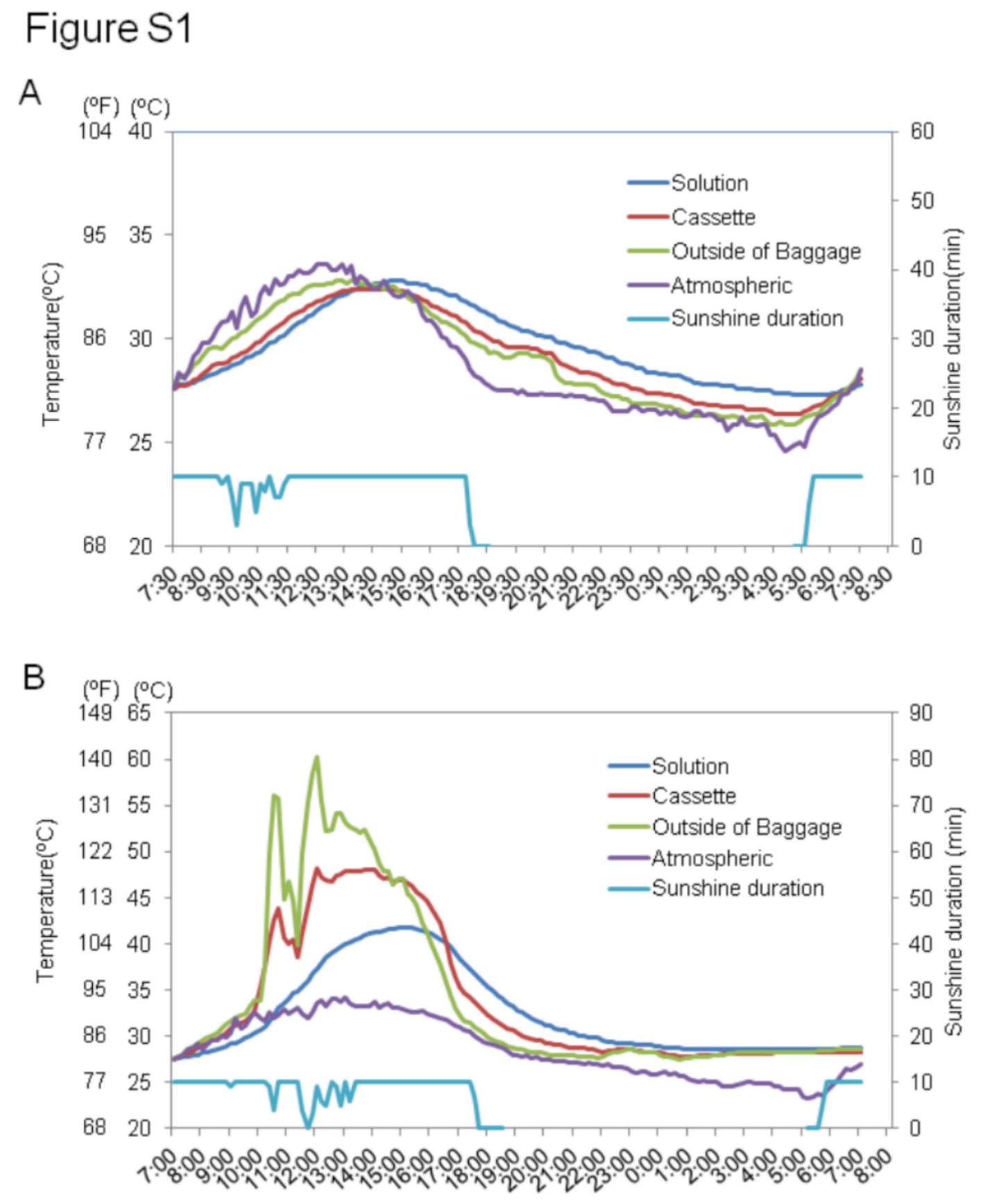

Supplement: Figure S1 — Temperature changes in (A) shaded and (B) sunny regions. Temperature changes outside the bag, around the cassette and in the solution. They were recorded when the bag was left outdoors in the shade (A) and in the sunny (B) areas. (TIF) [file pone.0052216.s001.tif]

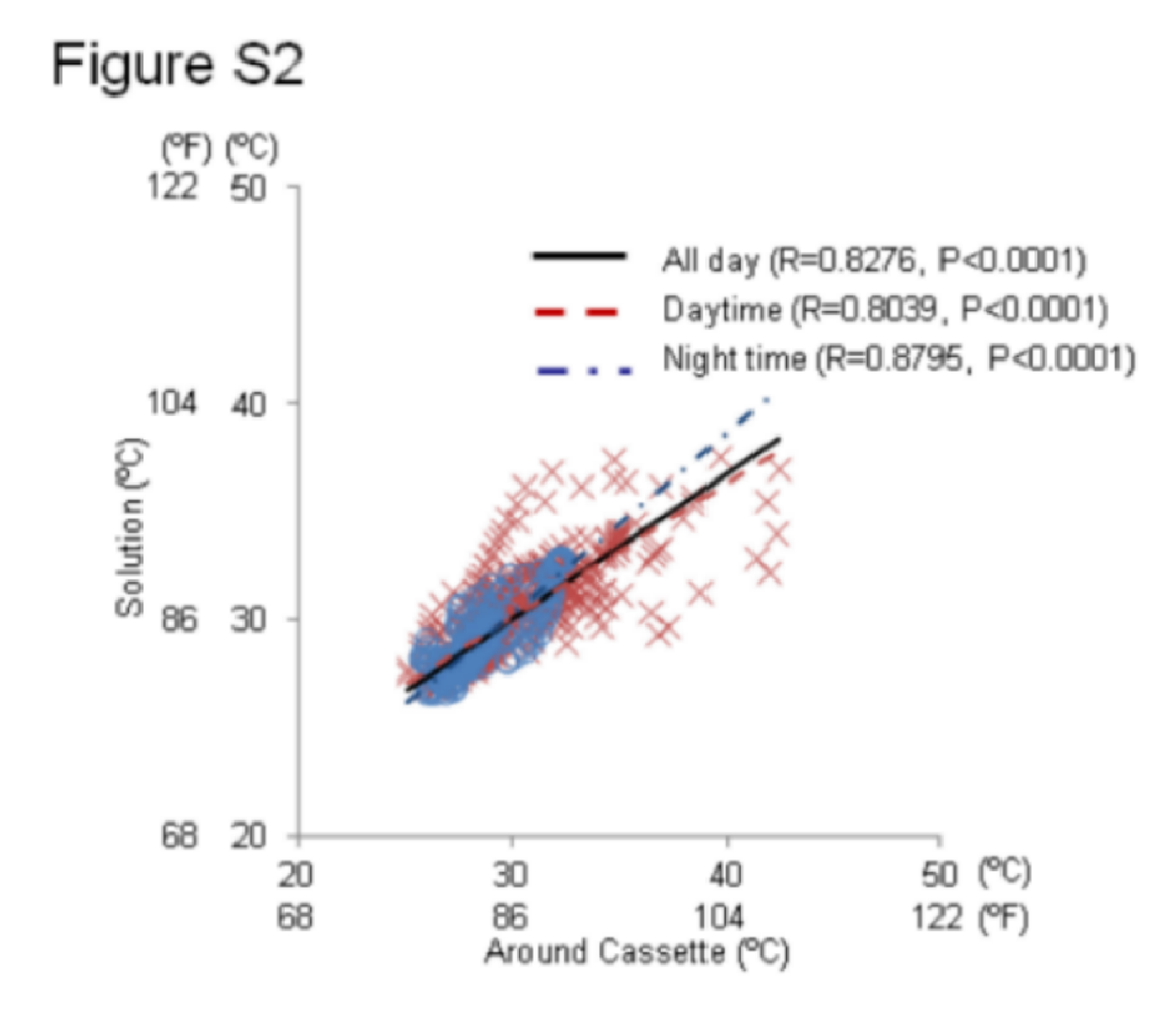

Supplement: Figure S2 — Temperature correlation between the solution and around the cassette. Correlations between daytime (×) and night (○) temperatures around the cassette and in the solution. The relativity of temperatures within groups was analyzed using the CORR procedure. Regression curves from plotted graphs were calculated using the GLM procedure. (TIF) [file pone.0052216.s002.tif]

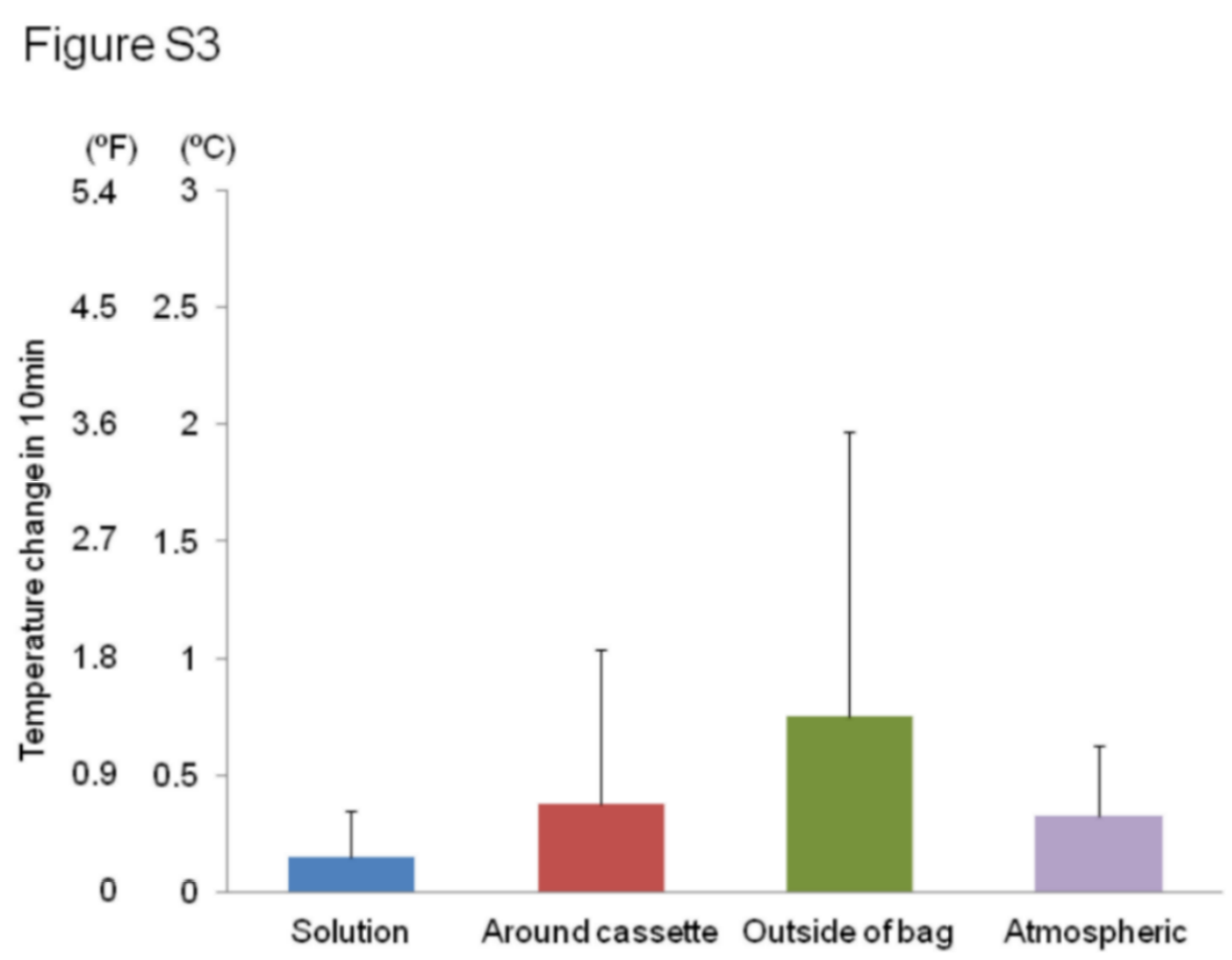

Supplement: Figure S3 — Mean temperature changes over time. Temperature changes every 10 minutes were analyzed in the solution, around the cassette, outside the bag, and in the atmosphere (0.15±0.19(SD)°C/0.27±0.34°F, 0.37±0.66°C/0.67±1.2°F, 0.77±1.25°C/1.38±2.26°F and 0.32±0.30°C/0.58±0.54°F, respectively). (TIF) [file pone.0052216.s003.tif]
