# Supplementary material for: Temperature Variations around Medication Cassette and Carry Bag in Routine Use of Epoprostenol Administration in Healthy Volunteers
Source: PLoS One. 2012 Dec 27;7(12):e52216. doi: 10.1371/journal.pone.0052216 (PMC3531421; doi:10.1371/journal.pone.0052216)
Supplement: Table S1 — Atmospheric temperature and duration of time in which the temperature was higher than 25, 35 or 40°C around cassette on determination of each sample. (DOC) [file pone.0052216.s004.doc]

Table S1. Atmospheric temperature and duration of time in which the temperature was higher than 25, 35 or 40ºC around cassette on determination of each sample

| Sample No | 1 | 2 | 3 | 4 | 5 | 6 | 7 | 8 | 9 | 10 | 11 | 12 | 13 | 14 | 15 | 16 |
| --- | --- | --- | --- | --- | --- | --- | --- | --- | --- | --- | --- | --- | --- | --- | --- | --- |
| Mean atmospheric temperature (ºC / ºF) | 30.5 / 86.9 | 30.9 / 87.6 | 29.2 / 84/.6 | 31.3 / 88.3 | 27.7 / 81.9 | 30.1 / 86.2 | 27.0 / 80.6 | 31.7 / 89.1 | 28.6 / 83.5 | 28.0 / 82.4 | 28.8 / 83.8 | 29.1 / 84.4 | 28.9 / 84.0 | 30.7 / 87.3 | 30.2 / 86.4 | 31.7 / 89.1 |
| Maximum atmospheric temperature (ºC / ºF) | 33.6 / 92.5 | 34.3 / 93.7 | 34.4 / 93.9 | 36.3 / 97.3 | 31.9 / 89.4 | 35.1 / 95.2 | 31.1 / 88.0 | 37.0 / 98.6 | 33.3 / 91.9 | 31.6 / 88.9 | 34.8 / 94.6 | 35.3 / 95.5 | 34.7 / 94.5 | 37.2 / 99.0 | 35.4 / 95.7 | 37.3 / 99.1 |
| Minimum atmospheric temperature (ºC / ºF) | 28.7 / 83.7 | 28.5 / 83.3 | 25.2 / 77.4 | 27.8 / 82.0 | 24.4 / 75.9 | 25.8 / 78.4 | 23.1 / 73.6 | 27.4 / 81.3 | 25.6 / 78.1 | 25.5 / 77.9 | 25.2 / 77.4 | 25.3 / 77.5 | 24.9 / 76.8 | 26.9 / 80.4 | 25.9 / 78.6 | 26.7 / 80.1 |
| Determination time ( minutes) | 1450 | 1450 | 1450 | 1450 | 1450 | 1450 | 1450 | 1450 | 1450 | 1450 | 1450 | 1450 | 1450 | 1450 | 1450 | 1450 |
| Cumulative time ( minutes) in which temperature was higher than 25ºC (77ºF) around cassette | 1450 | 1450 | 1450 | 1450 | 1450 | 1450 | 1450 | 1450 | 1450 | 1450 | 1450 | 1450 | 1450 | 1450 | 1450 | 1450 |
| Cumulative time ( minutes) in which temperature was higher than 35ºC (95ºF) around cassette | 0 | 40 | 70 | 0 | 0 | 20 | 100 | 210 | 0 | 250 | 30 | 60 | 0 | 0 | 170 | 600 |
| Cumulative time ( minutes) in which temperature was higher than 40ºC (103ºF) around cassette | 0 | 0 | 0 | 0 | 0 | 0 | 10 | 0 | 0 | 0 | 0 | 50 | 0 | 0 | 20 | 310 |
